# Automatic Direction of Spatial Attention to Male Versus Female Stimuli: A Comparison of Heterosexual Men and Women

**DOI:** 10.1007/s10508-015-0678-y

**Published:** 2016-02-08

**Authors:** Robert J. Snowden, Catriona Curl, Katherine Jobbins, Chloe Lavington, Nicola S. Gray

**Affiliations:** School of Psychology, Cardiff University, Cardiff, CF10 3AT UK; Department of Psychology, Pastoral Healthcare, Cardiff, UK; School of Medicine, Swansea University, Swansea, UK

**Keywords:** Sexual interest, Gender, Spatial attention, Dot-probe task

## Abstract

Abundant research has shown that men’s sexual attractions are more category-specific in relation to gender than women’s are. We tested whether the early automatic allocation of spatial attention reflects these sexual attractions. The dot-probe task was used to assess whether spatial attention was attracted to images of either male or female models that were naked or partially clothed. In Experiment 1, men were faster if the target appeared after the female stimulus, whereas women were equally quick to respond to targets after male or female stimuli. In Experiment 2, neutral cues were introduced. Men were again faster to female images in comparison to male or neutral images, but showed no bias on the male versus neutral test. Women were faster to both male and female pictures in comparison to neutral pictures. However, in this experiment they were also faster to female pictures than to male pictures. The results suggest that early attentional processes reveal category-specific interest to the preferred sexual category for heterosexual men, and suggest that heterosexual women do not have category-specific guidance of attentional mechanisms. The technique may have promise in measuring sexual interest in other situations where participants may not be able, or may not be willing, to report upon their sexual interests (e.g., assessment of paedophilic interest).

## Introduction

Motivationally salient stimuli are thought automatically to attract the allocation of cognitive resources (Yiend, 2010). Sexual stimuli might be thought among the most motivationally salient stimuli and empirical evidence supports their importance (Schimmack, 2005). Some theories of sexual response (Barlow, 1986; Janssen, Everaerd, Spiering, & Janssen, 2000) specifically include a process of attentional allocation to sexual images for further evaluation. Thus, an understanding of what stimuli automatically attract attention can be used to test theories of sexual attraction—for example, one might predict that spatial attention would be attracted to the location of an image of an attractive member of the opposite gender in heterosexual participants.

Recent research has shown stark differences in men’s and women’s pattern of sexual arousal to erotic stimuli. Using measures of sexual arousal via genital responses, it has been shown that men show a category-specific pattern of responses, with heterosexual men showing arousal to female erotic stimuli and homosexual men showing arousal to male erotic stimuli. On the other hand, several studies have shown that heterosexual women did not show this category-specific response and show significant arousal to both male and female erotic stimuli (Bossio, Suschinsky, Puts, & Chivers, 2014; Chivers, Rieger, Latty, & Bailey, 2004; Chivers, Seto, & Blanchard, 2007; Steinman, Wincze, Sakheim, Barlow, & Mavissakalian, 1981; Suschinsky, Lalumiere, & Chivers, 2009). The reasons for this lack of specificity are not clear. It has been suggested that this may reflect a protective mechanism that automatically prepares the genitals via lubrication and so reduce the chance of injury even under conditions where sexual activity is unwanted. It has been noted that such sexual responses have been noted when women view depictions of sexual coercion (Both, Everaerd, & Laan, 2003) which suggests that sexual arousal, at least as measured via genital vasocongestion, is initiated by sexual stimuli even for stimuli that depict coercion. An alternate hypothesis is that the non-specific response is an example of female sexual flexibility (Baumeister, 2000). This theory sees female sexuality as less tied to the biological needs and physical representation of a sexual partner or stimulus, but more defined via cultural or socioeconomic influences.

Sexual arousal is only a part of a sexual response. Sexual arousal to a stimulus involves a complex array of processes that include affective processes, cognitions, behavioral tendencies as well as physiological changes (Rosen & Beck, 1988). Recently, some of these other processes have been investigated with a view to examining the hypothesis that men show category-specific processes and women do not. Snowden and Gray (2013) adapted the Implicit Association Test (IAT) (Greenwald, McGhee, & Schwartz, 1998) to look at the association between male and female pictures and the concept of sex. They found that heterosexual men were fast and accurate when pictures of females and sex words were paired, and slow and inaccurate when pictures of males and sex words were paired, whereas heterosexual women did not show this category specificity (see also Camperio Ciani & Battaglia [2014] and Snowden, Wichter, & Gray [2008] for data on men). They further showed that this was not due to a lack of sexual associations in women, but that they show sexual associations to both male and female stimuli, whereas men only show this to female stimuli.

Other techniques have also examined possible gender differences in sexual interest. For instance, Israel and Strassberg (2009) used a viewing time paradigm (where the amount of time spent looking at a picture is recorded) to show that women looked at male pictures longer than men did, and that men looked at female pictures longer than women did. They also noted that while both men and women appear to show category-specific responses to the opposite sex, the size of this effect was substantially different, with men showing a large difference in viewing times (1069 ms) compared to women’s (220 ms). Imhoff et al. (2010), using a range of variations on the viewing time paradigm, consistently found categorical responses for both men and women. Rieger and Savin-Williams (2012) measured the size of the pupils while people watched video recordings of individuals masturbating. For heterosexual men, strong pupil dilation was found when viewing a female stimulus, but for heterosexual women strong pupil dilation was found for both male and female stimuli (see also Rieger et al., 2015). Hence, men consistently show stronger category specificity in relation to gender than women do.

The measures outlined above (IAT, viewing time, pupillary dilation) all tap into different aspects of the processing of a stimuli, such as the associations it forms in memory, and its arousing effects. As previously mentioned, automatic attention to sexually relevant stimuli is an important part of some models of sexual response (Barlow, 1986; Janssen et al., 2000) which appears to precede these later components (though it seems likely that connections will be reciprocal with associations in memory serving to help guide spatial attention, etc.). However, it is not yet known whether this early automatic spatial allocation of resources is gender category-specific for either men or women.

### Dot-Probe Task

**Fig. 1:**
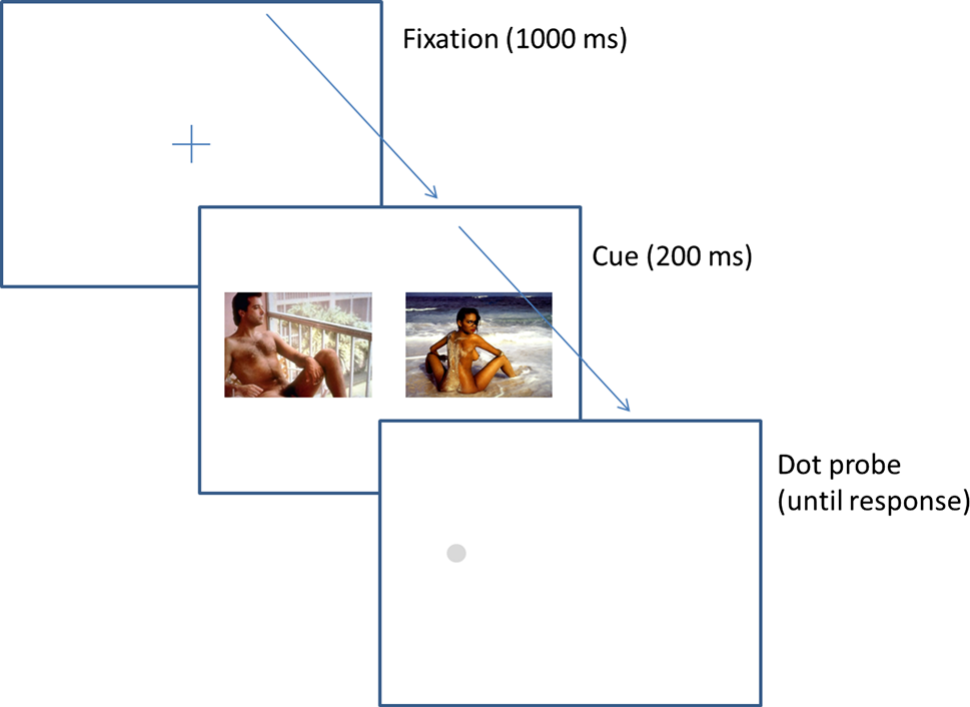
Depiction of events in a dot-probe trial

The dot-probe task is a well-established technique for examining attentional processes in cognitive psychology (see Bar-Haim, Lamy, Pergamin, Bakermans-Kranenburg, & van Ijzendoorn [2007], and Frewen, Dozois, Joanisse, & Neufeld [2008] for reviews). The paradigm is illustrated in Fig. 1. In this paradigm, a fixation point is first presented. Then, two stimuli, termed the cues, are presented in unison for a short period of time at equal distances from the point of fixation. The two cues, typically, consist of a test cue (for example, an angry face) and a control cue (for example, a neutral face). These stimuli are then removed and a target stimulus (typically a small dot) appears at the location of one of the previous cues. Participants then respond to this target by signaling either its presence, location, or form. It is well established that responses to stimuli that are presented at the location where visual attention has already been located are faster and more accurate than those at other locations (e.g., Nakayama & Mackeben, 1989; Posner, 1980; Snowden, Willey, & Muir, 2001). Hence, if one of the two cues summons visual attention more effectively than the other, we should expect probes at this location to be processed faster than at the other location and reaction times (RTs) to the probe should be smaller (or measures of accuracy should show fewer errors).

Several studies have demonstrated that participants are faster and more accurate for targets that follow certain classes of cue in comparison to neutral cues. For example, stimuli that induce fear, such as a snake, produce shorter RTs when the dot is presented after this cue than if presented after the neutral cue (Lipp & Derakshan, 2005; Ohman, Flykt, & Esteves, 2001; Van Damme, Crombez, & Notebaert, 2008), and that the extent of these biases is sensitive to individual differences and clinical conditions (e.g., Macleod, Mathews, & Tata, 1986; Townsend & Duka, 2001). Hence, the dot-probe task appears to be sensitive to attentional biases and could be used to measure such biases to sexual stimuli.

There have been two studies that have looked at whether sexual stimuli per se attract attention in comparison to neutral stimuli using the dot-probe task. According to the logic presented above, a sexual stimulus would be expected to attract attention, and targets that follow at this location should enjoy an advantage over the opposing location. Prause, Janssen, and Hetrick (2008) performed such an experiment. However, the results were surprising in that the detection of the target dots was slower following a sexual cue compared to the neutral cue, and that this effect was greater in people reporting higher sexual desire. Further, this “reversed” effect was greater for stimuli with more explicit sexual content (such as images of penile-vaginal intercourse and oral sex) in comparison to less explicit images (e.g., nudes). There were no effects of gender on these results. One possible explanation for this pattern of results is that the sexual stimuli produce a grabbing of attention that also ties up processing for some time, hence, there are not enough resources to process the dot stimuli or for decision making (Geer & Bellard, 1996; Wright & Adams, 1994). The situation may be analogous to an “emotional Stroop” effect whereby the emotional content of the stimulus is processed with greater priority than other aspects of the image (Bourke & Gormley, 2012; Ciardha & Gormley, 2012; Gress, Anderson, & Laws, 2013). It is notable that Wright and Adams (1994) also found that the detection of a dot within a sexual picture (rather than *after* a sexual picture) was found to be slower for images containing their preferred sex, and this was found for both men and women that were either heterosexual or homosexual. More recently, Kagerer et al. (2014) examined individual differences to images of sex (containing both a man and a woman in each image) as opposed to neutral images in a dot-probe a paradigm, with a cue to target interval of 500 ms. In contrast to the data of Prause et al. (2008), Kagerer et al. show the participants were faster to targets that followed the sexual image, though the effect was small (10 ms). Men showed an effect of 14 ms (which was significant), while women showed an effect of 5 ms (which was not significant). However, this difference between the genders was not statistically significant.

In studies of spatial cueing, a distinction is often made between exogenous (also known as automatic or reflexive) and endogenous (also known as deliberative or voluntary) movements of attention (Posner, Grossenbacher, & Compton, 1994). Exogenous attention is short-lived and is usually tested by having cues that are not predictive of the target’s location (i.e., the target was presented at the location of the cue on 50 % of trials and on the opposite side to the cue on 50 % of the trials, hence the presentation of the cue does not give any further information about the likely location of the target) and using short cue to target intervals (≤200 ms). Endogenous attention is usually tested by having cues that are predictive of the target’s location (i.e., the target was presented at the location of the cue on, for example, 80 % of trials and on the opposite side to the cue on 20 % of the trials, hence the presentation of the cue does give information about the likely location of the target) and using longer cue to target intervals (≥500 ms)—(Nakayama & Mackeben, 1989; Tales, Muir, Bayer, & Snowden, 2002). The study of Prause et al. (2008) used cues that were not predictive of target location but had a relatively long cue to target interval (500 ms). Indeed, an interval of 500 ms appears to be the most commonly used in dot-probe studies in general. However, 500 ms (as used by Kagerer et al. and Prause et al.) is at the outer limits of the time normally associated with exogenous movement of attention to simple cues (Müller & Rabbitt, 1989; Nakayama & Mackeben, 1989), and may allow for non-automatic, or controlled, processes to contribute. Hence, it is unclear what aspects of attention may have been engaged in their experiment. As we are most interested in the present study in the automatic effects of the presentation of a sexual stimulus, we sought to provide greater isolation of this exogenous component by using cues that were not predictive of target location and had a short cue to target interval (200 ms).

We could find no studies that have used the dot-probe task to compare responses when male and female stimuli are used as the two cues—the studies of Prause et al. (2008) and of Kagerer et al. (2014) used sexual images that contained both male and females in each image. On the basis of the theories of heterosexual sexual interest, we predicted that men would be faster to probes following a female cue, whereas women would show no difference in speed to probes that followed either male or female cues.

## Experiment 1

Based on the idea that men are category-specific in their sexual interests, we predicted this would be reflected in the automatic allocation of attention, and they would be faster to target that occurred at the location of the cue of their explicitly stated preferred stimuli (in this case female cues as all participants were heterosexual). Our second hypothesis was that this effect would not be found for women as these automatic processes that allocate attention are not closely related to their explicitly stated sexually preferred stimulus.

### Method

#### Participants

Participants were recruited from a large urban UK university. We deliberately recruited a greater sample of women as we predicted that we would get large effect sizes for the men, but smaller effect sizes for the women and therefore wanted greater power in this latter group. Recruitment occurred via posters and advertisements for volunteers to take part in an experiment on human sexuality. All participants were undergraduate students of age 18 or older (age was not measured) and were given course credit for this participation.

A total of 73 (52 women, 21 men) were recruited. Eight (six women, two men) of the participants reported a non-heterosexual orientation (scores of 2 or greater on the 0–6 scale on the Kinsey scale (Kinsey, Pomerory, & Martin, 1948)) and their data were excluded from all analyses. Data from the dot-probe task were lost due to software error for six female participants. Finally, two participants were excluded (one woman, one man) due to high error rates (>25 %) on the dot-probe task. We report, therefore, data from 39 women and 18 men in the dot-probe task.

#### Measures

Eight male and eight female pictures, from the International Affective Picture System (Lang, Bradley, & Cuthbert, 1997) were selected. Each picture featured one person either nude or partially clothed but not performing any sexual act. The male pictures were IAPS 4460, 4470, 4490, 4500, 4520, 4534, 4550, and 4561. The female pictures were 4002, 4003, 4141, 4142, 4210, 4232, 4235, and 4240. The pictures were approximately matched in terms of poses and actions and were all chosen to be sexually attractive in pilot studies.

All stimuli were presented on a computer screen (refresh rate 60 Hz) and were viewed from approximately 57 cm. All experiments were conducted in a sound-attenuated laboratory with a low level of background luminance. All experiments used the DirectRT programme to present the stimuli and record the responses of the participants.^1^

##### Ratings of Sexual Attractiveness

Before the main experiment each participant made ratings of sexual attractiveness for each picture on a scale of 1 to 5 (1 = *very sexually appealing*, 3 = *neither appealing nor unappealing*, 5 = *very sexually unappealing*). Stimuli were presented one at a time without time constraints and the participants typed their response into the keyboard. Data presented are reversed-scored so that higher scores represent more attraction to the image.

##### Dot-Probe Task

The dot-probe task required the participant to signify the location (left vs. right) of a small faint test dot (1 cm diameter, gray approximately 40 cd/m^2^) on a white background (approximately 80 cd/m^2^) that appeared after the cueing pictures. A faint target was used as this has been shown to produce greater sensitivity to manipulations of attention (Snowden et al., 2001).

Figure 1 illustrates a sample trial. Each trial commenced with a fixation cross (1000 ms) in the middle of the screen. This was followed by the cue stimulus (200 ms). The cue always consisted of two images, one of a female and one of a male, each centered 12 cm from the middle of the screen. The cue was then replaced with the test stimuli (the dot) which was also centered 12 cm from the middle of the screen and remained until a response was made.

To construct cue images of male–female pairs, we first matched each male picture to one female picture that was most similar in terms of pose and race of the images. This pair was used to make one cue with the male on the left and female on the right, and one cue with this order reversed. Hence, we produced 16 cues in this manner. We then matched each male picture to the next most similar female picture, and produced another 16 cues in the same manner, so that we had a total of 32 cues. These 32 cues were used to construct a block of 64 trials (dot on either the left of right location for each cue) which were presented in random order. A second block was then completed for a total of 128 trials. RTs and errors were recorded.

##### Measure of Sex Drive

The sex-drive questionnaire (SDQ: Ostovich & Sabini, 2004) was used to assess the participant’s strength of sex drive. The brief four-item questionnaire asks about sexual activities that do not require a partner (experiencing sexual desire, number of orgasms, frequency of masturbation, and self-rating of sex drive). Responses were recorded via a 7-point Likert-type scale.

#### Procedure

Participants were recruited via advertisements around the University that asked for volunteers for an experiment on human sexuality. On arrival at the laboratory, participants read an information sheet that explained that the experiments would involve viewing and reacting to pictures and words of a sexual nature. They were asked if they wished to preview the stimuli before the main experiment (though no participant requested to do this). The nature of the questionnaires was explained and that all data would be held anonymously. They had the opportunity to ask questions and then signed a consent form. Participants then completed the questionnaire measures (the SDQ, position in menstrual cycle, self-reported sexual orientation) and the Kinsey scale. The dot-probe task was one of the three tasks that was administered as a battery and was administered last, while the other two tasks were an IAT and a priming task that utilized the same images as the dot-probe task. The results of the IAT and the Priming task replicated previous findings (Snowden et al., 2008; Snowden & Gray, 2013) and so are not reported here but are available from the corresponding author. At the end of testing, the participants were thanked, given a debrief form that explained the purpose of the experiments, and were given their course credits.

#### Data Analysis

The raw RT data were trimmed by the removal of trials with RTs less than 200 ms or greater than 2000 ms. Data from two participants were removed due to excessive (>25 %) error rates. We performed two analyses on the data. The first used “traditional” methods to examine the RT data (see, for example, Mogg et al., 2000). First, trials in which the person incorrectly located the target (errors) were removed (mean error: *M* = 2.1 %, *SD* = 2.8, range = 0–12.2 %). Mean RT was then calculated separately for the targets following male pictures and then for the female pictures.

This “traditional” method of analysis suffers from a number of problems. First, increased speed in one condition (or for one individual) may come at the cost of increased errors (a speed-accuracy trade-off). If error trials are simply removed from the analysis (as they most often are due to the relatively small percent of error trials) important information related to possible speed-accuracy trade-off is lost. Second, there are large individual variations in even simple RTs. Hence, a particular change, let us say 100 ms, in RTs may reflect a large percentage change for someone with fast RTs, but a smaller percentage change for someone with slow RTs. Greenwald, Nosek, and Banaji (2003) have proposed a series of scoring algorithms for use with a particular RT paradigm, the Implicit Association Task, which combines both errors and RTs and tries to account for individual differences in overall speed. We, therefore, used some aspects of these algorithms to produce a “D-score” for our second analysis. First, error trials were not removed, but were penalized by adding 600 ms (which is approximately the average RTs in our experiments) to the actual RT. We then calculated the D-score by calculating the difference in RTs between the two conditions (targets following male or following female pictures) and dividing this by the pooled SD from each condition. This D-score is analogous to a z-score in that it expresses the difference in units of standard deviation for each person. Positive D-scores, in our calculation, indicate faster reactions to targets located after the female picture.

### Results

#### Ratings of Attractiveness

At a global level, the male and female pictures did not differ in their overall ratings (female = 3.05, male = 2.70; *t*(54) = 1.62, *p* = .11). As expected, men rated the female pictures as more sexually attractive (Female = 4.34, Male = 1.33; *t*(17) = 23.99, *p* < .001), while women rated the male pictures as more sexually attractive (Female = 2.54, Male = 3.17; *t*(38) = −3.55, *p* < .001).

#### Sex Dot-Probe Task

Before testing our hypotheses, we first examined the reliability of the dot-probe task, as several authors have suggested that other versions of the dot-probe task are unreliable (Dear, Sharpe, Nicholas, & Refshauge, 2011; Kappenman, Farrens, Luck, & Proudfit, 2014; Schmukle, 2005; Staugaard, 2009). Reliability was calculated from split-half (using even and odd trials) correlations of the difference scores between mean RTs for targets following male versus female cues, this was then corrected by the Spearman–Brown prophecy formula (Cunningham, Preacher, & Banaji, 2001). Reliability was moderate for the total sample (*r* = .55) and for the female sample (*r* = .66), but was somewhat lower for the male sample (*r* = .29)

**Fig. 2:**
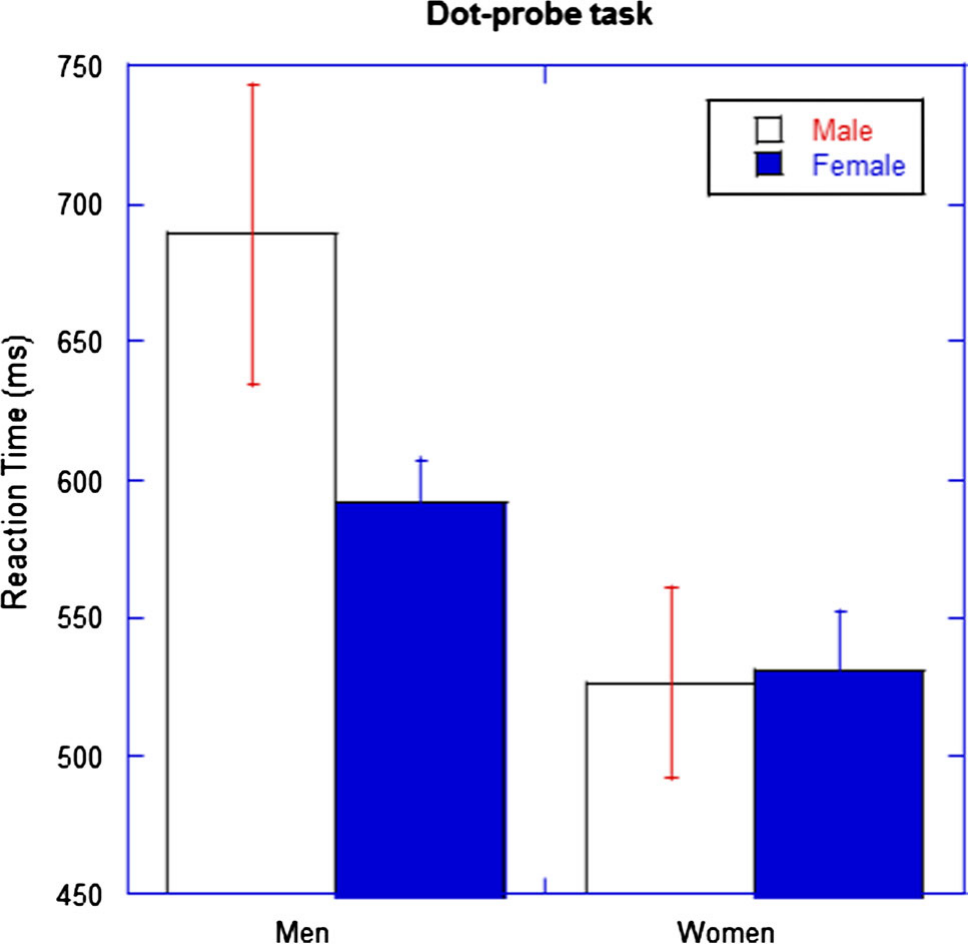
Data from Experiment 1. Mean reaction times (ms) from the dot-probe task are plotted for men and women. The open columns are for targets following male picture cues, and the filled columns are for targets following female picture cues. *Error bars* represent ± 1 standard error of the mean

Figure 2 illustrates the results, using the traditional RT measure, for participants to detect the target after it appeared at the location of a previous male or previous female cue. A two-way ANOVA with factors of cue (male vs. female) and gender of participant (women vs. men) was performed. There was a significant main effect of cue, *F*(1, 55) = 21.88, *p* < .001, *η*_p_^2^ = .285, and of gender, *F*(1, 55) = 7.14, *p* = .01, *η*_p_^2^ = .115. There was also a significant interaction between these variables, *F*(1, 55) = 8.30, *p* = .006, *η*_p_^2^ = .131. Planned comparisons showed that men were slower when the target appeared after a male rather than female cue (614.9 vs. 568.4 ms; *t*(17) = 4.71, *p* < .001; *d* = 2.28), while there was no effect of cue for women (510.8 vs. 499.9 ms; *t*(38) = 1.58, ns).

Using the D-scores we found that the men had a positive score (*M* = 0.249; SE = 0.041) which was significantly different from 0, *t*(17) = 6.11, *p* < .001, while the D-score for women did not differ from 0, (*M* = −0.048, SE = 0.049: *t*(38) = 0.97, ns).

#### Sexual Drive and Dot-probe task

As expected, men reported greater levels of sex drive on the SDQ, *t*(55) = 5.09, *p* < .001; *d* = 1.28. However, the SDQ did not significantly correlate with the D-score on the dot-probe task for either men (*r* = .05, ns) or for women (*r* = 0.09, ns).

### Discussion

The results of experiment 1 were entirely in line with our hypotheses—heterosexual men were faster for targets that appeared at the location that followed the female cue rather than the male cue. Women, on the other hand, did not show different responses for targets occurring after the male or the female cues.

One notable feature of our results related to the reliability of the test. Previous reports on the dot-probe task found low levels of reliability (Dear et al., 2011; Kappenman et al., 2014; Schmukle, 2005; Staugaard, 2009). We suggest that the relatively poor reliability of those studies may well be related to the rather small (or possible non-existent) effects that they were trying to detect. Clearly, if there is not an effect to measure (or a very small one), the experimenter is left with only the “noise” of the paradigm and the instrument will appear unreliable. However, when there is a signal that has considerable variation between people, then the instrument’s reliability will increase as this signal’s power rises against the error noise. Hence, in the present experiment, we get good reliability for the dot-probe task as we have strong individual differences in sexual interest.

It might be suggested that the difference in men and women is related to their differences in sexual drive (Wehrum et al., 2013) rather than any gender specific factor. In our experiment, however, we did not find any relationship between our measure of sex drive and the dot-probe effect to this male versus female cue comparison. Kagerer et al. (2014) did report significant correlations, for both men and women, in the magnitude of their sex versus not-sex dot-probe effect to some self-report measures of sexual interest, and suggested that differences in overall sex drive might account for the smaller dot-probe effect in females. However, we note that (1) their experimental paradigm measures general interest in sex, as they compare sex cues to neutral cues, whereas the present study compares male cues to female cues; (2) the difference due to gender in the dot-probe effect reported by Kagerer et al. was not significant; (3) several of the measures of sex drive used by Kagerer et al. were not significantly related to the dot-probe effect; (4) the measure most similar to that used in the present study (the Sexual Desire Inventory-Solitary measure: Spector, Carey, & Steinberg, 1996) did not find a significant correlation with the dot-probe effect for either men (*r* = −.02) or women (*r* = 0.10). Hence, we do not think that the present results can be explained by differences in levels of sexual drive in men and women.

## Experiment 2

Experiment 1 appears to establish that heterosexual men show an automatic allocation of attention to images of the opposite gender, whereas heterosexual women do not. However, the pattern of results does not tell us the reasons as to why women did not show faster performance to targets following the male cues. One possible reason is that such pictures simply fail to produce a spatial attention effect in women. It is reported that women tend to have lower interest in sex than men do (e.g., Alexander & Fisher, 2003; Dewitte, 2015; Oliver & Hyde, 1993) and this might be reflected in less automatic attention to sexual stimuli. Hence, both the male and the female stimuli might have failed to reach a threshold whereby spatial attention was summoned. The second possibility is that both the male and female sexual stimuli attracted attention, but that this was of approximately equal strength and, therefore, there was no overall measurable differential effect. This idea would be in accord with our main hypothesis that heterosexual women are approximately equally attracted to males and females at this automatic level.

Experiment 2 aimed to test between these possibilities by introducing images that contained no sexual content. We used cues that pitted male against female pictures as in Experiment 1, but we now also compared male cues to neutral cues, and female cues to neutral cues, in separate tasks. We predicted that men would show faster responses for targets following the female cues in comparison to male or neutral cues, but would show no difference in the male versus neutral condition. Women, on the other hand, should show no bias in the male versus female condition, but would show a bias to the male and to the female cues when presented in comparison to the neutral cues.

### Method

#### Participants

A total of 91 (48 women, 43 men) participants, recruited under the same conditions as Experiment 1, completed the experiments. Eight (seven women, one man) of the participants reported a non-heterosexual orientation (scores of 2 or greater on the 0–6 scale on the Kinsey scale (Kinsey et al., 1948)) and their data were excluded from the analyses. Finally, three participants were excluded (one female, two male) due to high error rates (>25 %) on the dot-probe task. We, therefore, report data from 40 women and 40 men.

#### Measures

Most of the procedures were identical to those from Experiment 1, and only the changes are highlighted here.

The main change was the inclusion of neutral stimuli. For half the participants (20 women, 20 men) these were also chosen from the IAPS and were images of natural scenes, objects, and man-made objects (IAPS numbers: 5220, 5260, 5300, 5390, 5660, 5873, 7000, 7030). We did not attempt to match these neutral images to the images of males and females on such dimensions as valence and arousal, as, by their very nature, we expect the sexual images to differ on these dimensions. We did attempt to choose images that matched approximately in term of complexity. However, our interest was in whether we get a different pattern of results for men and women and the complexity of the images would be the same for both men and women. Cues for the neutral versus male task were produced in the same manner as described for the male versus female cues in Experiment 1. First, each of the male images was paired with one of the neutral pictures, and two cues were produced with the male image on the left for one cue and with it on the right for the other cue. Hence, 16 cues pairs were produced. This was then repeated with the each male picture now paired to a different neutral image, so that we had 32 cues. The same process was used to produce 32 female–neutral cues.

The second major change was that for half the participants we used images that were altered to remove the background. In these altered images, we only presented the foreground figure (subjectively defined by ourselves), and replaced the background with a solid blue of approximately the mean luminance of the image. This was done in an attempt to control for possible differences in the salience of the background stimuli and therefore the visibility of the main focus (foreground) of the picture. For some of the neutral images the “foreground” was not well-defined, so we selected a new set of images that all contained pictures of mushrooms (sourced from the internet). These images were resized to match the male and female pictures, and the background was replaced by the same blue background as for the male and female slides. In the preliminary analysis of these data, we could find no main effect or interactions that were due to the two sets of stimuli. Hence, we collapsed all data across this manipulation.

The experiment consisted of 192 trials: 64 contained male versus female cues, 64 contained male versus neutral cues, and 64 contained female versus neutral cues. All locations of cues and targets were counter-balanced. The 192 trials were presented in a random order that differed for each participant.

Finally, given the lack of any significant effects for the measures of sexual drive in Experiment 1, we did not take a measure of sexual interest or drive in Experiment 2.

### Results

**Fig. 3:**
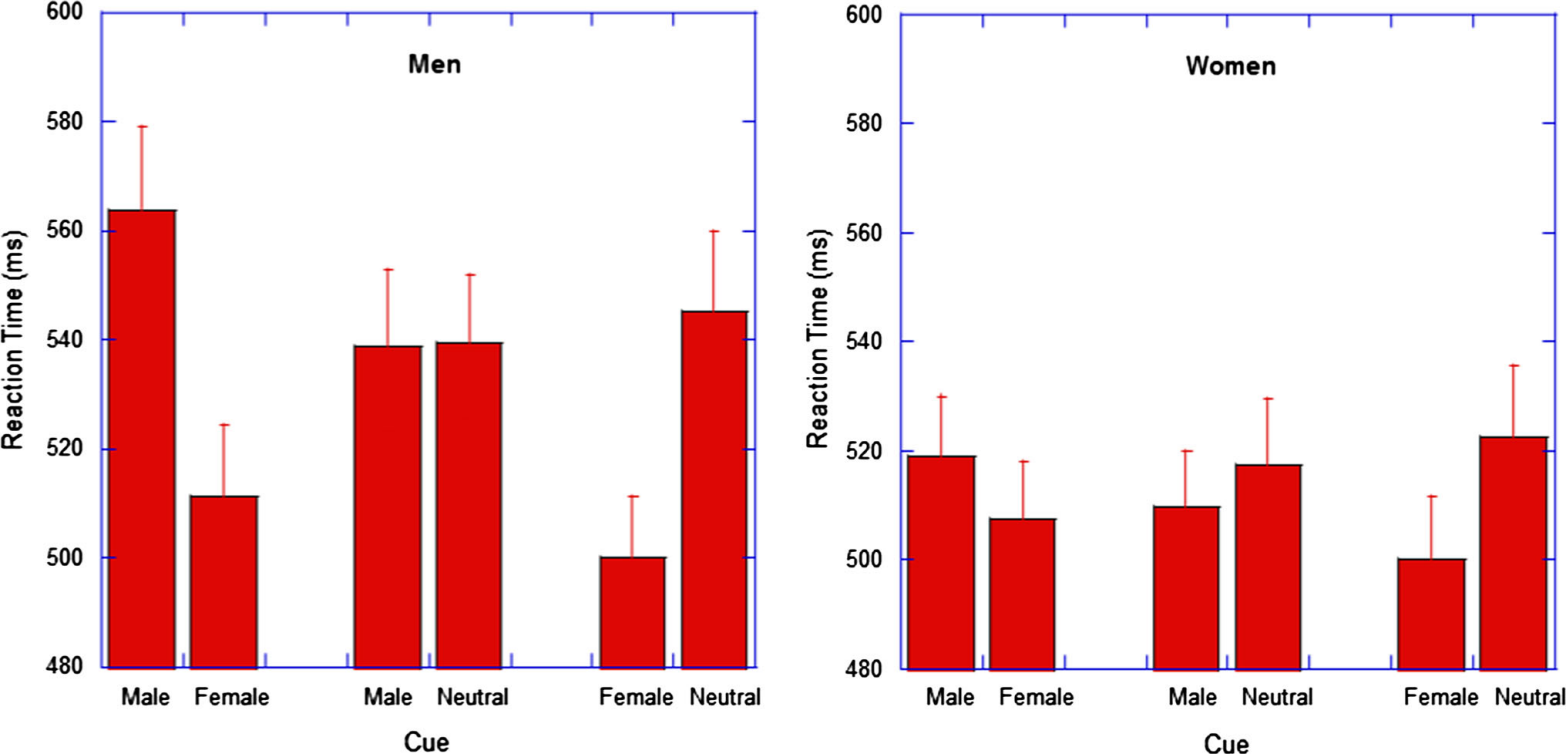
Data from Experiment 2. Mean reaction times (ms) from the dot-probe task are plotted for men (*left section*) and for women (*right section*). *Error bars* represent ± 1 standard error of the mean

The reliability for each of the comparisons was tested via split-half reliability corrected with the Spearman–Brown formula. For the female–male comparison the reliability was .38 (men; *r* = .48, women: *r* = .08), for the male–neutral comparison it was .10 (men; *r* = −.44, women: *r* = .48), and for the female–neutral comparison it was .54. (men; *r* = .54, women: *r* = .45),

Figure 3 illustrates the RT results from the three conditions (Fig. 3a for men and Fig. 3b for women). A two-way 6 × 2 ANOVA with factors of cue type^2^ (male–female; female–male; male–neutral, neutral–male; female–neutral; neutral–female) and gender of participant (women vs. men) was performed. There was a significant main effect of picture, *F*(5, 390) = 28.36, *p* < .001, *η*_p_^2^ = .267, but not of gender, *F*(1, 78) = 1.27, *p* = .26, *η*_p_^2^ = .016. There was a significant interaction between these variables, *F*(5, 390) = 6.16, *p* < .001, *η*_p_^2^ = .073.

#### Male Versus Female

To understand this interaction, we performed a series of planned comparisons. As in Experiment 1, for the male versus female trials, men were faster when the target appears after the female cue (563.5 vs. 511.5 ms; *t*(39) = 5.56, *p* < .001; *d* = 0.91). For the women, we also found faster performance when the target appeared after the female cue (524.1 vs. 507.2 ms; *t*(39) = 3.41, *p* = .002; *d* = 0.57).

#### Male Versus Neutral

For the male versus neutral comparison, we found no effect for men (538.6 vs. 539.2 ms; *t*(39) = 0.16, *p* = .87; *d* = 0.02), whereas, for the women, there was a trend toward faster responses following the male stimuli (509.6 vs. 517.4 ms; *t*(39) = 1.38, *p* = .08; *d* = 0.22).

#### Female Versus Neutral

For the female versus neutral comparison, we found a strong effect for men to respond faster to targets after the female cue (500.1 vs. 545.4 ms; *t*(39) = 6.72, *p* < .001; *d* = 1.20). Women also responded faster to targets after the female cue (500.2 vs. 522.3 ms; *t*(39) = 3.55, *p* = .002; *d* = 0.57).

#### *D*-Scores

We also analyzed the *D*-scores: a two-way ANOVA with levels of comparisons (male vs. female, male vs. neutral, and female vs. neutral) and gender showed a main effect of comparison, *F*(2, 156) = 17.93, *p* < .001, *η*_p_^2^ = .187, and of gender, (*F*(1, 78) = 11.73, *p* = .001, *η*_p_^2^ = .131. There was a significant interaction between these variables, *F*(2, 156) = 6.96, *p* = .001, *η*_p_^2^ = .082.

Planned comparisons showed that men showed significant positive scores for the female–male comparison (*D* = 0.329; *t*(39) = 7.83, *p* < .001), and the female–neutral comparison (*D* = 0.372; *t*(39) = 8.31, *p* < .001), but showed no bias for the male–neutral comparison (*D* = 0.021; *t*(39) = 0.69, *p* = .49).

Women showed positive scores on the female–male comparison (*D* = 0.133; *t*(39) = 3.96, *p* < .001), and the female–neutral comparison (*D* = 0.168; *t*(39) = 3.78, *p* = .001) but also showed a significant positive score on the male–neutral comparison (*D* = 0.076; *t*(39) = 1.79, *p* = .04).

### Discussion

Our hypotheses were partially supported by Experiment 2. For the men, the data were entirely as predicted. The dot-probe task showed a strong attentional bias to female pictures when paired with either male pictures or with neutral pictures. On the other hand, there was no indication of a bias when male pictures were paired with neutral pictures. We save further discussion of these results for the main discussion.

For women, the hypotheses were only partially fulfilled. They showed a strong bias toward female pictures in comparison to neutral pictures (though smaller than for the men), and a small bias toward male pictures in comparison to neutral pictures. However, in the female–male comparison, we found a greater bias toward the female pictures than the male pictures (not the bias toward male stimuli that one might intuitively predict for heterosexual females). This latter result is somewhat different to that of Experiment 1 that showed no bias. We note, however, that in experiments using other indirect measures of sexual associations that biases toward female stimuli have also been found in heterosexual females (Snowden & Gray, 2013).

The reliability estimates for the tasks were, on the whole, comparable to Experiment 1 and typical of indirect measures of cognitions (Fazio & Olson, 2003). The notable exception to this was the reliability of the male–neutral test in the men. Here, the measure of internal consistency was negative! We can offer no explanation for this result.

## General Discussion

The usual interpretation of the dot-probe paradigm is that the two cues compete for attention. If one stimulus gains more attention processing then resources are given to this location and RTs to targets at this location will be faster. We chose a cue duration that was short (200 ms) as this short duration is thought to exclude any use of deliberative processes (Müller & Rabbitt, 1989). Hence, we suggest that this shift of attention to the preferred image involves an automatic process, such as those envisaged in theories such as that of Janssen et al. (2000), that focuses attention resources onto the salient target for further evaluation and action. Our results show that men tend to allocate their attention preferentially to the female stimuli, whereas women showed equal attention to the male and female stimuli (Experiment 1) or even a bias toward female stimuli (Experiment 2). As such, our results support the idea that heterosexual men show a category-specific response to sexual stimuli, in this case via spatial attention to women, whereas heterosexual women show no evidence of category specificity toward men. To our knowledge, there are no other studies that have used this technique of using cues of a single sex in order to compare men and women on this task.

As discussed in the Introduction, other researchers have used a dot-probe type task that compared stimuli of a sexual nature (e.g., a couple kissing) against those of a non-sexual nature (Prause et al., 2008). In light of the results we present here, one might predict that attention would be attracted to the sexual stimuli. However, the pattern of results of Prause et al. was just the opposite. It was suggested that this may be due to a second effect where a sexual stimulus not only summons attention to itself but then continues to hold this attention, slowing responses to stimuli following a sexual cue. Such an explanation is supported by the data of Wright and Adams (1994) who showed that people are slower to find a target dot hidden on a picture of their preferred gender than on their non-preferred gender. The data of Prause et al. also show some other interesting features. First, the “reversed dot-probe” effect was greatest for the stimuli that were the most explicit in terms of sexual content. However, Kagerer et al. (2014) used images with two levels of explicitness, but did not report any differences in the results for these images of sex versus not-sex. The images used in the present experiment would be regarded as low in explicit sexual content in comparison to the images used by Prause et al. and some of those used by Kagerer et al. It seems possible that stimuli that are more explicit might produce a different pattern of results. For example, it could be that men have a lower threshold for allocating attention to sexual images. Hence, the stimuli we used were able to trigger this process for men, but not for women—though we note that the stimuli were able to cause attentional allocation when paired against neutral stimuli. Further work might vary the level of sexual explicitness from weak to strong to test if allocation of attention remained category-specific for men but not for women.

While we attempted to use stimuli that were sexually attractive to the opposite sex, the ratings show that we were only partially successful. Men did judge the female pictures as more sexually attractive than the male pictures, and women did judge the male pictures as more sexually attractive than the female pictures. However, the magnitude of the difference between the male versus female ratings was far greater for the men than for then women. In other words, men were far more categorical in their judgements than were the women. Hence, it could be argued that the “driving force” behind the dot-probe effect was weaker for women than it was for the men and this might therefore explain the null result for women. This “confound” is probably present in many other studies of sexual attraction that use stimuli of a single sex. Indeed, many studies have shown that women give more similar ratings of attractiveness to both males and females, while men tend to give very different ratings (Bradley, Codispoti, Sabatinelli, & Lang, 2001), which probably reflects the greater importance men place on physical attractiveness (Feingold, 1990). Hence, these “explicit” ratings and the “implicit” findings of the present experiment and others (e.g., Snowden & Gray, 2013) both show strong categorical effects for men toward their preferred sex, but weak effects for women.

### Limitations and Future Research

We believe the present results provide strong evidence that the dot-probe task can reveal important information about how a person views a particular sexual stimulus—i.e., if this stimulus strongly attracts attention. The dot-probe task might, therefore, be used to look at possible deviant sexual interests and be added to the growing number of such tasks that could be used for clinical and research purposes (see Snowden, Craig, & Gray, 2011). However, a number of issues would need to be addressed before the adoption of the dot-probe task as a clinical/forensic instrument (see below).

The dot-probe task has some desirable properties compared to other indirect methodologies. The response required is simply the detection of a small target stimulus and does not require any specific language skills, or the need to categorize sexually related targets such as images or words. This means the task can be performed by those with poor verbal intellectual abilities, limited vocabulary, or limited reading skills (which are common in offender populations). Hence, it may allow testing of the development of sexual interest in children and infants, or the examination of deviant sexual interests in juvenile sexual offenders. It could also be easily adapted to the study of sexual interest in other species, allowing for parallel research in humans and other species.

In the present experiments we attempted to isolate the fast, reflexive, automatic components of attention. Further research is needed to see if more deliberative cognitive processes (or endogenous attention) produce similar results and what the implications of this would be. Indeed, the parameters we used in our experiments were somewhat arbitrary (based on information from research in other topics) and parametric studies are needed to optimize this task for this particular area of research, and to extensively examine the psychometric properties of the task such as its test–retest reliability, resistance to faking, etc. This is important as indirect measures of cognition tend to show only moderate reliability. In the present experiments, internal reliability was generally moderate but was low (and even reversed) for the male–neutral task.

Our task required the person to indicate the location of the target, which has been common practice in dot-probe tasks. However, this may not be ideal for two reasons. First, the cue might not only cause a shift of attention (and hence better stimulus processing) but may also cause a response bias to respond in this direction (of course, for the present experiments even if this were the case it would support the arguments being made about the distribution of attention). Second, the task could be solved by the realization that the target was *not* at the location being examined, so no further shift of attention is required. Future experiments might use a judgement of stimuli identity that is orthogonal to the location of the target so that this target must be processed. Experiments examining the issue of whether these hypothesized shifts of attention produce changes in sensitivity or bias are also warranted (Van Damme et al., 2008).

Finally, we have presented our task as one measuring an automatic component of sexual interest. Theoretically, this dot-probe task measures whether spatial attention is allocated more to the female or male (or neutral) image but does not tell us the reason for this allocation. It is not hard to imagine other possible reasons for allocating attention (e.g., novelty, fear, disgust) that may also contribute to the present results. As an example, a person might find the representation of male genitalia disgusting and therefore produce a repulsion away from such a stimulus—this would “appear” as an attraction to female stimuli in this paradigm and could contribute to the finding that heterosexual women were faster for targets located after the female cue compared to the male cue. Some women might also be more interested in the female pictures for purposes of “social comparison” (Festinger, 1954; Tiggemann & McGill, 2004). At this point in time, we are not able to rule out such alternate theories for the dot-probe task, but note that similar critiques could be applied to paradigms such as viewing time (Israel & Strassberg, 2009) or pupil response (Rieger & Savin-Williams, 2012).

### Conclusions

The current experiments tested the notion that heterosexual men and women have different patterns of sexual interest by comparing their performance on a dot-probe task that pitted images of attractive males and females against one another, or against neutral images. As hypothesized, men showed a category-specific response indicative of sexual attraction toward their explicitly stated preferred gender, whereas women did not. Our results show broad support for the notion that heterosexual women do not show category-specific sexual attraction to their explicitly preferred gender choice.

## Footnotes

1 One reviewer has suggested that the use of a keyboard to record RTs limited our ability to detect differences due to its poor temporal resolution. However, Ulrich and Giray (1989) show that this is not a problem for experiments such as that presented here.

2 The cue name refers to the two cues presented, and the target dot appears at the location of the first cue name.
